# Magnetic Characteristics of FeSiB Cores in Motors Revealed by Experiment and Finite-Element Simulation

**DOI:** 10.3390/ma18102325

**Published:** 2025-05-16

**Authors:** Meng Wang, Long Hou, Wenwei Ju, Yan Ma, Zhongkai Guo, Dianguo Ma, Lanju Liang, Haishun Liu, Weiming Yang

**Affiliations:** 1 School of Opto-Electronic Engineering, Zaozhuang University, Zaozhuang 277160, China; zzxygdwm@163.com (M.W.); guozhongkai123@163.com (Z.G.); madianguo@163.com (D.M.); lianglanju123@163.com (L.L.); 2 School of Mechanics and Civil Engineering, China University of Mining and Technology, Xuzhou 221116, China; 15252019190@163.com; 3 School of Materials Science and Engineering, Anhui University of Technology, Ma’anshan 243002, China; 18365342025@163.com; 4 School of Materials and Physics, China University of Mining and Technology, Xuzhou 221116, China; liuhaishun@126.com

**Keywords:** FeSiB cores and loss, motor, magnetic flux density, finite-element simulation

## Abstract

Iron core loss (*P*_cm_) is the main source of energy dissipation in motors, primarily affected by the stator material, which necessitates the optimization of soft-magnetic materials. In this work, the magnetic characteristics of FeSiB amorphous alloys and their influence on motors were systematically investigated via both experiment and finite-element simulation. It was found that the *P*_cm_ of the FeSiB core initially decreased significantly during heating but subsequently increased with a further temperature rise. In particular, after annealing at 460 °C for 10 min, the FeSiB core exhibited the lowest *P*_cm_ of 0.11 W/kg (50 Hz, 1 T) and 5.45 W/kg (1 kHz, 1 T), which correlated well with the changes in the magnetization. With the help of the finite-element analysis, the low *P*_cm_ of the motor using the FeSiB core was further demonstrated, and was closely associated with the dominance of the stator loss. Additionally, the magnetic flux density cloud and the related electromagnetic torque of the motor were comparatively analyzed to unveil the potential advantages of the current FeSiB core. This work provides an important theoretical basis for the design and development of amorphous/nanocrystalline motors.

## 1. Introduction

Under the background of the carbon emissions peak and carbon neutrality, the energy crisis and the continued growth of electrical power generation need to minimize their wasteful energy dissipation and maximize the conversion efficiency of the electronic power devices that are used [1,2,3]. As the largest consumer of electricity, the motor has become one of the key aspects of national energy conservation and emission reduction work, with its energy dissipation mainly originating from iron core loss (*P*_cm_) [3]. The soft-magnetic iron core is one of the key factors that determine the loss and efficiency of the motor [4,5]. Currently, electrical steels, mainly composed of iron (Fe) with less than 6.5 wt.% silicon (Si), remain the predominant materials used in applications such as transformers and motors owing to their relatively low cost and high saturation magnetization (1.8–2.0 T). However, electrical steels are hindered by high-frequency core loss caused by their inherently low electrical resistivity (48–82 µΩ·cm) [6,7]. Inversely, Fe-based amorphous soft-magnetic alloys, rendering tremendous interest, have become the focus of academia and industry due to their wide range of applications in motors and transformers by virtue of their low loss characteristics at high frequencies [8,9,10]. Sun et al. reported the influence of stators made of different magnetic materials on the efficiency of a high-speed asynchronous motor and found that the *P*_cm_ can be significantly reduced by 91.1% using a stator made of an FeSiB amorphous alloy in 0–800 Hz [11]. Liu et al. studied the magnetic field distribution, stator core loss, and efficiency of two motors and demonstrated that amorphous material improved the electromagnetic performance of low-speed motors, and also exhibited higher efficiency than that of a silicon steel motor with an increasing frequency [5]. Gao et al. verified that by replacing the silicon steel stator with the amorphous metal stator and improving the motor design, the efficiency of the motor can be increased by 4.76%, and the weight can be reduced by 11.36% [12]. Although extensive studies about motors using soft-magnetic amorphous alloys have been reported [13,14,15,16], the performance and process optimization of amorphous magnetic cores, as well as their compatibility with motor design, are still a long-term issue that needs to be explored urgently.

In this work, a typical FeSiB amorphous alloy and silicon steel as the stator materials were selected for comparison, and the influences of the stator material on the magnetic characteristics including core loss, magnetic flux density, and electromagnetic torque were systematically investigated via experiment and finite-element simulation.

## 2. Materials and Methods

Commercially available amorphous ribbons with a nominal composition of Fe_80_Si_9_B_11_ (at.%) were purchased from Jiangsu Guoneng Alloy Technology Co., Ltd. Jiangsu, China. The purity of the raw materials, including iron (Fe), silicon (Si), and boron (B), was 99.9 wt%. As-spun ribbons with widths of approximately 15 mm and thicknesses of approximately 28 μm were produced using the single roller melt-spinning method, which were then wound into round cores with an outer diameter of 28 mm, an inner diameter of 20 mm, and a height of 15 mm. Heat treatment was performed by keeping the ribbons or iron cores in a quartz tube under a vacuum atmosphere (10^−3^ Pa), and then pushing the quartz tube into a tubular furnace preheated to annealing temperature (420–480 °C) for various lengths of time, followed by water quenching cooling.

The microstructures of the air-sides of specimens were verified by X-ray diffraction (XRD, Bruker D8 Discover, Bruker AXS, Karlsruhe, Germany) with Cu *Kα* radiation and transmission electron microscopy (TEM; Talos F200X, FEI, Hillsboro, OR, USA). Here, for the XRD test, the scanning angle range is 20–90°, and the step size is 0.02°. Thermal behaviors were confirmed by differential scanning calorimeter (DSC, NETZSCH 404 C, Netzsch, Selb, Germany) with a heating rate of 0.33 °C/s under high-purity Ar protection. Similar to the previous works [17,18], the as-quenched ribbons were cut into 6 mm and then isothermally annealed in the vacuum chamber (10^−3^ Pa) to prepare the annealed ribbons. The magnetic properties of ribbons including B-H curves, maximum magnetic flux density (Bs), and coercivity (Hc) can be obtained by a vibrating sample magnetometer (VSM, EZ7, MicroSense, Lowell, MA, USA) under an applied field of 800 kA/m and a B-H loop tracer (BHS-40, RIKEN Machinery, Tokyo, Japan) under a field of 1 kA/m in DC mode, respectively. Moreover, the core loss and magnetization curves of the wound cores were measured via a silicon steel measuring instrument (MATS-3000M, Ningbo Economic and Technological Development Zone Kainuo Instrument Co., Ltd., Jinan, China) and soft-magnetic AC measuring device (MATS-2010SD, Lianzhong Technology Co., Ltd., Beijing, China), respectively. The electrical resistance was measured using a conventional DC four-probe method (SZJG ST2742B, Suzhou Jingge Electronics Co., Ltd., Suzhou, China). The finite-element simulation on the electromagnetic properties of motors using different soft-magnetic materials as stator components was carried out using ANSYS Maxwell software (ANSYS Electronics 2019 R3).

## 3. Results and Discussion

### 3.1. Microstructure and Thermal Behavior

**Figure 1:**
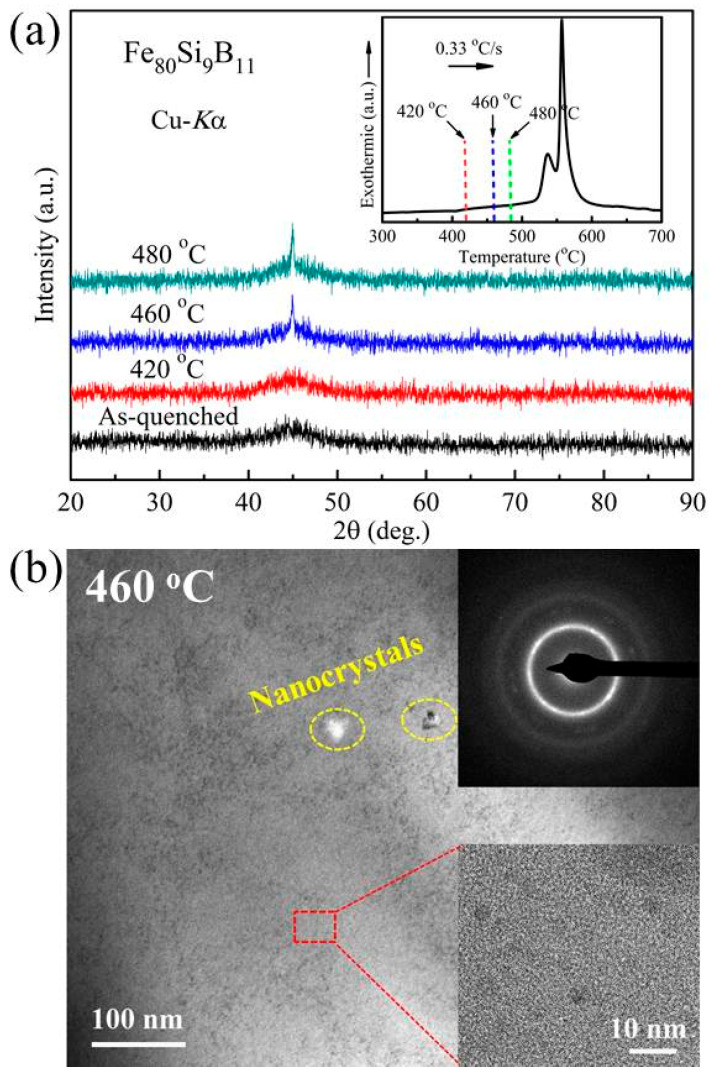
(**a**) XRD patterns of the Fe_80_Si_9_B_11_ amorphous ribbons annealed at different times for 10 min. The inset is the DSC curve of the as-quenched alloy. (**b**) TEM image of the Fe_80_Si_9_B_11_ alloy subjected to annealing at 460 °C for 10 min. The inset (**top**) shows the selected area electron diffraction (SAED) pattern, and the (**bottom**) shows this in relation to the local enlarged image.

Figure 1a shows the XRD patterns of the FeSiB amorphous alloys which are subjected to annealing for 10 min at different temperatures, corresponding to the temperatures just below the first exothermic peak in the DSC curve (shown in the inset in Figure 1a). With the temperature rising, a slightly sharp peak on a broad diffuse diffraction peak in the vicinity of 45° can be detected. Some works have elucidated that this change is assigned to the (110)-reflection of α-Fe crystalline phase and/or the formation of the clusters of crystal nuclei [19,20,21], which favors the optimized magnetic softness of the post-annealed alloys. It should be noted that this is different from the annealing temperature and time, which are 653 K and 60 min in Fe_80_Si_9_B_11_ [22]; the annealing temperature in the current work is relatively high for probing into the microstructure evolution. Furthermore, the microstructure of the present FeSiB alloy subjected to annealing at 460 °C for 10 min was illustrated using the TEM test, as shown in Figure 1b. A small number of nanocrystals can be identified on the amorphous matrix, revealing a slight crystallization, which coincides well with the XRD results. From the selected area electron diffraction pattern in the inset (top), there exist some bright dots in the diffraction ring. This is the typical characteristic of the (110)-reflection of α-Fe nanocrystals [23]. The local enlarged HRTEM image is given, as also shown in the inset (bottom). Some heterogeneous regions (crystal-like ordered clusters) are observed. These heterogeneities may act as the heterogeneous sites of α-Fe nanocrystals for the alloy during the post-annealing. Such amorphous nanocrystals and their transitional microstructures endow the alloy with potentially excellent magnetic properties.

### 3.2. Magnetization

**Figure 2:**
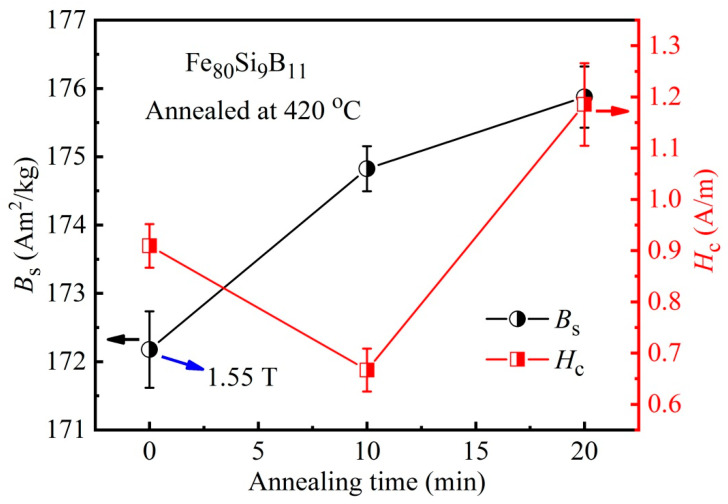
Variations of *B*_s_ and *H*_c_ with respect to the annealing time for Fe_80_Si_9_B_11_ amorphous ribbons after annealing at 420 °C.

**Figure 3:**
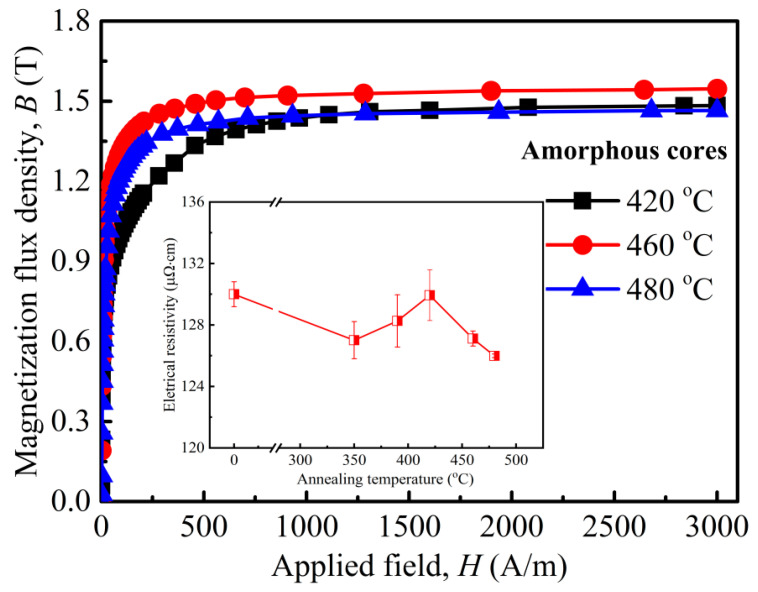
Compared magnetization curves of amorphous cores annealed at various temperatures for 10 min. The inset shows the electrical resistivity changes.

Figure 2 exhibits the magnetic softness of an example alloy after annealing at 420 °C for different annealing times. One can see that the lower *B*_s_ (172 Am^2^/kg) and larger *H*_c_ (0.91 A/m) in the as-quenched state are obtained, which is very close to the *B*_s_ of 1.56 T reported by the amorphous ribbon manufacturer [24]. With the annealing time increasing, the *H*_c_ drops to 0.67 A/m (10 min) and then increases, while *B*_s_ increases continuously, implying structural changes in the alloy after a shorter annealing time at a higher temperature. Based on this, the magnetic responses of the wound amorphous cores after annealing at different temperatures were analyzed, as shown in Figure 3. A regular magnetization curve for each amorphous core can be found, and the magnetization quickly saturates at a low applied field, implying good permeability and magnetic softness. The difference is that the amorphous core after annealing at 460 °C exhibits a quicker magnetization process and possesses a larger *B*_s_ of 1.54 T than that of 1.49 and 1.47 T at 420 °C and 480 °C, respectively, meaning a fast magnetic response of the present soft-magnetic material, which matches well with the fast start of the motor and other magnetic electronic devices [1,16]. Moreover, the inset in Figure 3 displays the changes in the electrical resistivity (*ρ*_e_) of annealed amorphous cores. One can see that the *ρ*_e_ of cores initially increased and then decreased, and the maximum value of *ρ*_e_ is measured to be 130 μΩ·cm at 420 °C.

### 3.3. Amorphous Core Loss

**Figure 4:**
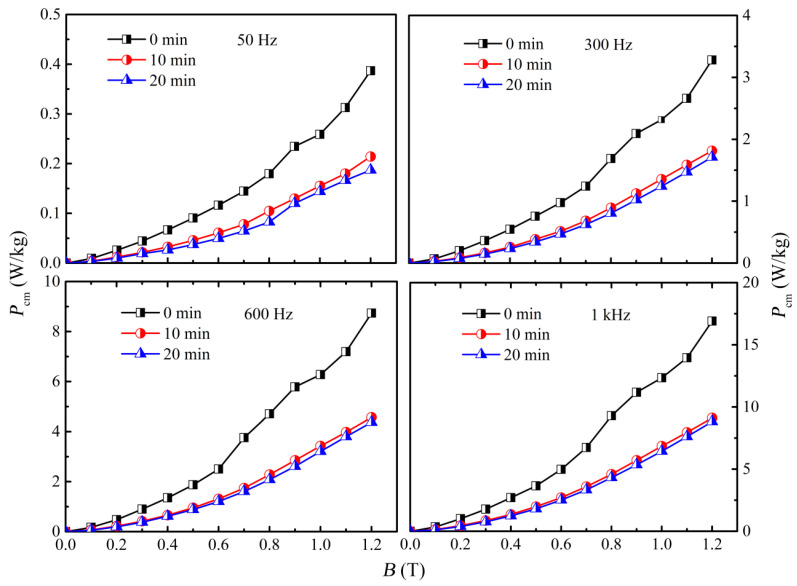
Dependence of loss in amorphous cores annealed at 420 °C on annealing time at various frequencies.

Furthermore, according to the lower *ρ*_e_ of the annealed amorphous core at 420 °C, the comparative *P*_cm_ of the amorphous cores annealed at 420 °C for various times is shown in Figure 4. It can be clearly seen that the *P*_cm_ displays a similar trend at the different frequencies, which declines compared to the as-quenched state but seems to be equal for 10 and 20 min. Moreover, similar to the phenomenon in other amorphous cores, the *P*_cm_ manifests a strong dependence on frequency (*f*) and *B*_m_. According to the classical loss separation model, the total iron loss can be described as follows [11,12,25]:

$$P_{cm}=P_{hyst}+P_{ec}+P_{r}=k_{h}fB_{s}^{2}+k_{e}f^{2}B_{s}^{2}+k_{ex}f^{1.5}B_{s}^{1.5}$$

where, *P*_hyst_, *P*_ec_, and *P*_r_ are hysteresis loss, eddy current loss, and residual loss, and *k_h_*, *k_e_*, and *k_ex_* correspond to the coefficients of hysteresis, eddy current, and residual loss, respectively. It is apparent that each loss component exhibits a different sub-exponential relationship with frequency (*f*) and *B*_s_, but both support an increase in *P*_cm_. Apart from *f* and *B*_s_, the *H*_c_ and resistivity (*ρ*_e_) should be highlighted. For instance, the *P_hyst_* is ∝ *B*_s_*H*_c_ [26]; *P_ec_* $\propto B_{s}^{2}/\rho_{e}$ [27]; and *P_r_*$\;\propto B_{s}\;f/\rho_{e}$ [8]. It can be clearly seen that compared to the as-quenched amorphous core, the annealed ones at 10 and 20 min show a lower *P*_cm_, especially at 20 min, implying the influence of microstructural changes in amorphous cores after annealing relaxation on *P*_cm_. As a result, taking into account the magnetization and *ρ*_e_ features, the 420 °C and 10 min were determined as the optimal parameters for the further exploration of the annealing process.

**Figure 5:**
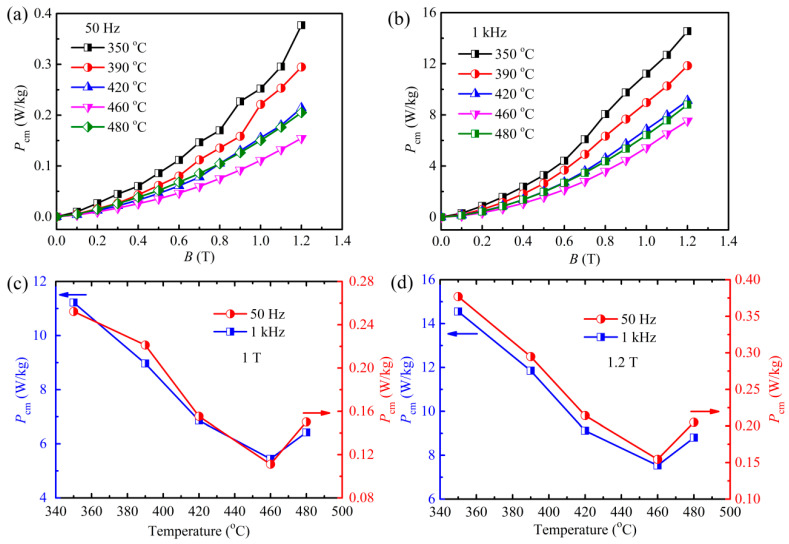
(**a**,**b**) loss of the amorphous core annealed at various temperatures as a function of the magnetic induction at 50 Hz and 1 kHz, respectively; (**c**,**d**) changes in the loss dependence on the various temperatures under an applied field of 1 T and 1.2 T, respectively.

To gain a deeper understanding of the correlation between *P*_cm_ and structural changes, the *P*_cm_ of amorphous cores after annealing at various temperatures is investigated, with the results shown in Figure 5. One can see that at both 50 Hz and 1 kHz, *P*_cm_ significantly drops as the annealing temperature increases from 350 °C to 460 °C, and then increases at 480 °C; similar trends are evident in Figure 5a,b. The comparative *P*_cm_ at 1 and 1.2 T as a function of the temperature is further extracted, as shown in Figure 5c,d. The *P*_cm_ declines first and then increases as the temperature rises. In particular, at 460 °C, the lower *P*_cm_ values of 0.11 W/kg at 50 Hz and 5.45 W/kg at 1 kHz are recorded at a magnetic field of 1 T. When the magnetic field is increased to 1.2 T, the *P*_cm_ increases to 0.16 W/kg at 50 Hz and 7.54 W/kg at 1 kHz under the same conditions. These values are still at the minimum across all temperature ranges, indicating the optimal microstructure at the current annealing temperature for amorphous cores.

**Figure 6:**
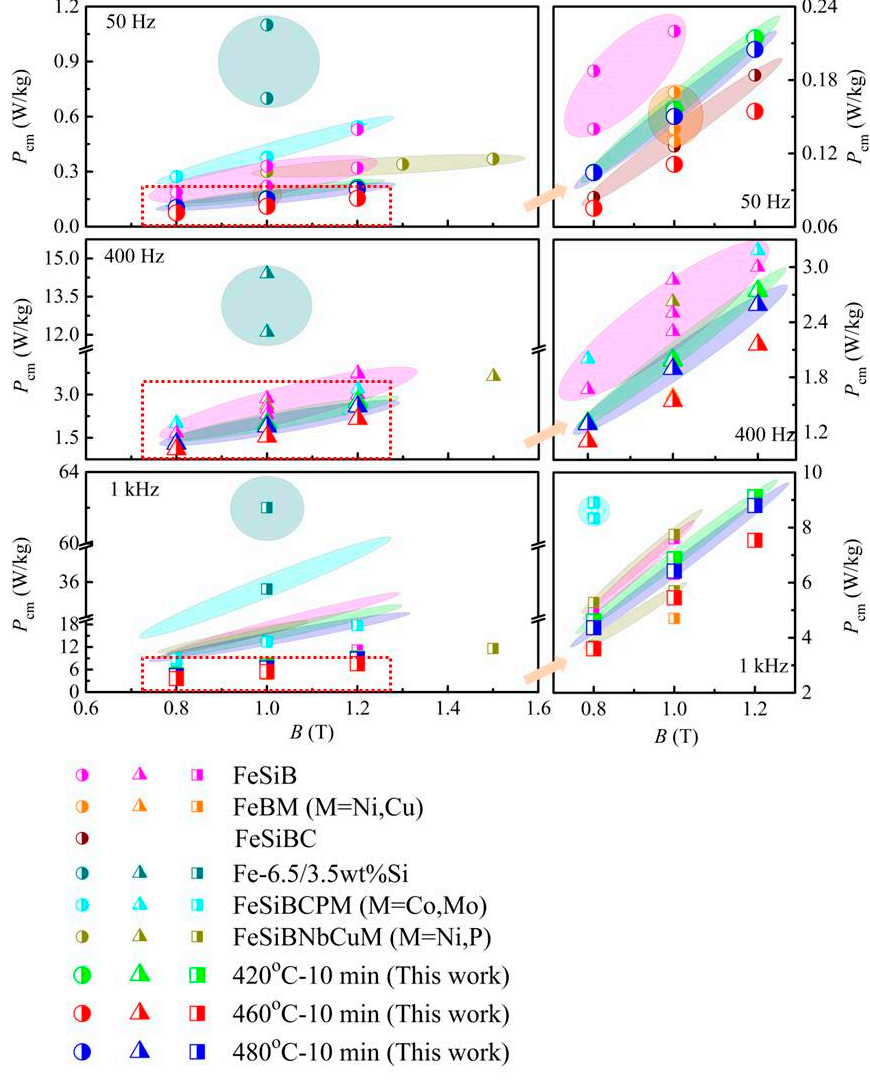
Relationship between core loss and magnetic flux density of cores wound by present alloys and some other reported amorphous alloys at different frequencies.

Next, the dependence of *P*_cm_ on magnetic flux density *B* for the current work (highlighted by the larger symbols in green, red, and blue) is compared with that of other FeB-based amorphous and Fe-Si crystalline alloys, as reported in references [15,25,28,29,30,31,32,33,34], and is shown in Figure 6. The figure on the right is a locally enlarged image for the rectangle dotted line area in the left figure. As can be seen, the *P*_cm_ of the displayed Fe-based amorphous alloys is much lower than that of the Fe-Si alloy, unveiling the unique magnetic properties and great application advantages of the former. Particularly, compared with the previous reports, the *P*_cm_ in the current work appears to be the lowest value at the same *f* and *B*, manifesting a better heat treatment process.

### 3.4. Finite-Element Simulation

**Figure 7:**
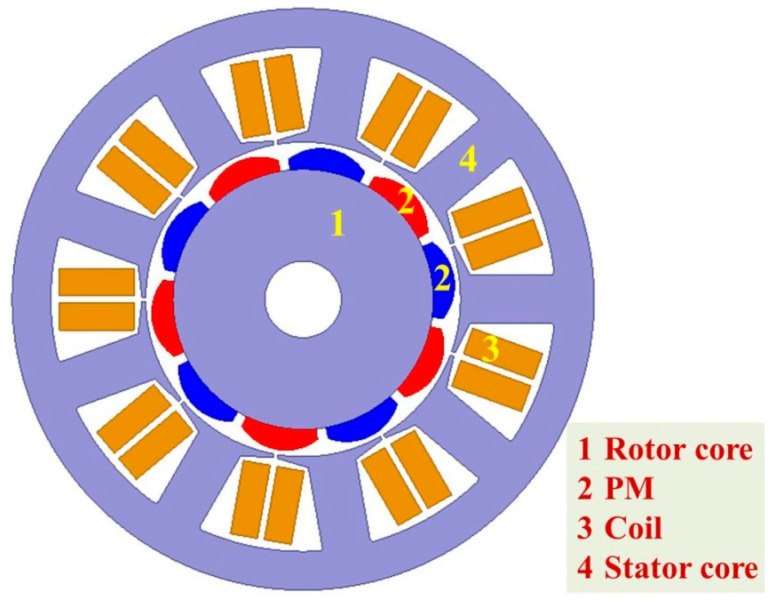
Motor construction diagram.

To accurately represent the real-time performance of the present amorphous cores as a stator component, the finite-element simulation model was utilized to analyze the loss, magnetic flux density distribution, torque, and efficiency of the motor during operation. In this work, a permanent magnet synchronous motor with a rated power of 750 W is employed, and the motor model is designed in Figure 7, which mainly involves four parts: rotor core, permanent magnet (PM), coil, and stator core. The detailed parameters are outlined in Table 1.

**Figure 8:**
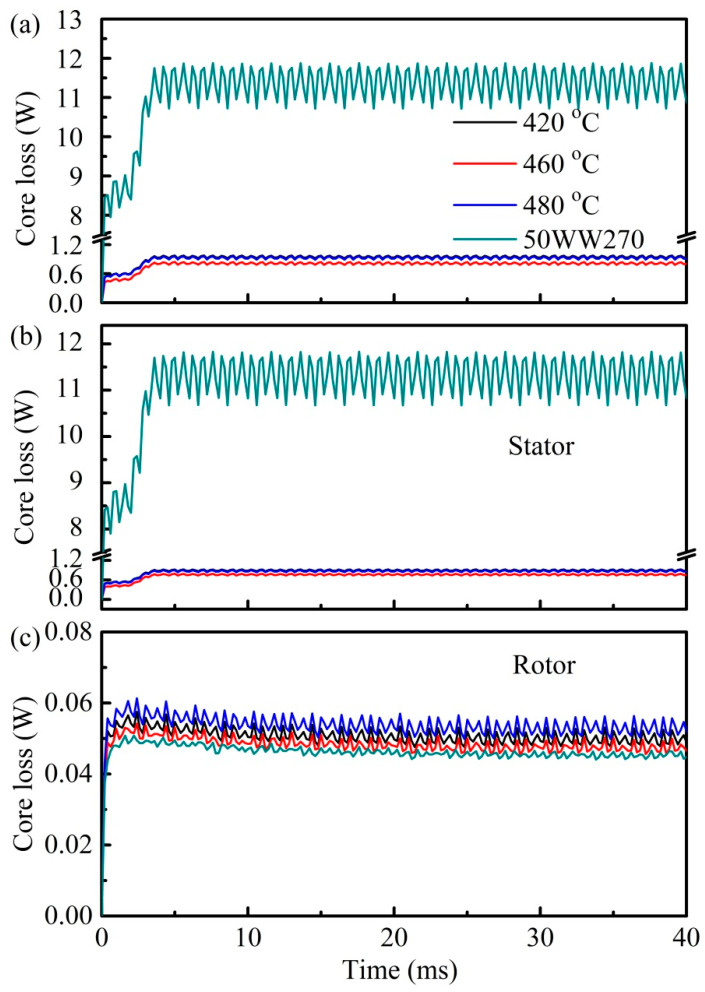
Core loss variations during motor operation: (**a**) total loss, (**b**) loss of stator, (**c**) loss of rotor.

Figure 8 exhibits the core loss distribution of a simulated motor operating at a rated speed of 3000 rpm. Also, the data of a typical silicon steel (SS, 50WW270) are shown for comparison. As shown in Figure 8a, the current amorphous core exhibits significantly lower core loss, approximately 1/10 to 1/14 of SS, which is crucial for power electronic devices and represents a distinctive advantage that is not observed in other soft-magnetic materials. This result is also consistent with the previous works reported in FeSiBC, FeSiBPCu, and FeSiBNbCu amorphous/nanocrystalline alloys, where the core loss is 1/2 to 1/5 smaller than that of Fe-Si alloy [3,35]. Moreover, the tiny difference in loss spectra for the present annealed alloys can be discerned, where the lowest loss for the amorphous core annealed at 460 °C is confirmed. This is in accordance with the above result in Figure 5. In addition, the loss of the separate stator and rotor are extracted from the total loss spectra, as shown in Figure 8b,c. It can be observed that the stator loss shows the same tendency and values as the total loss, while the rotor loss is significantly lower, being only approximately 1/200 of the total loss, and thus can be considered negligible in the current motor design. This unveils the dominance of stator losses in the overall loss profile and further implies the importance of designing soft-magnetic materials tailored to the stator in motor design. It is noted that the larger fluctuations in the loss of SS after stable operation can likely be attributed to the rapid increase in loss with the magnetization increase. However, when subjected to the minor influence of the present loss on magnetization, it manifests a small fluctuation. All of the above further confirm the superiority of the current soft-magnetic material for the stator design.

**Figure 9:**
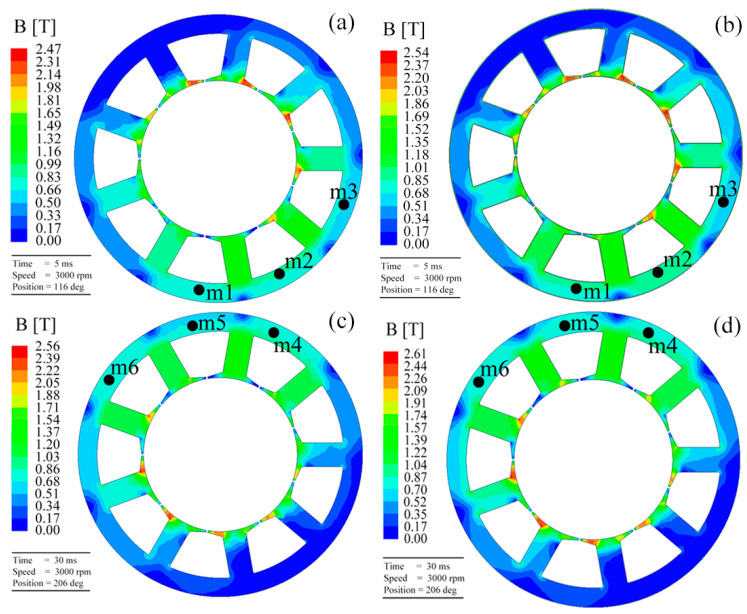
Magnetic density cloud of the motor using different soft-magnetic alloys for different running times at the rated operating conditions: (**a**,**c**) iron core annealed at 460 °C for 5 ms and 30 ms, respectively; (**b**,**d**) SS (50WW270) for 5 ms and 30 ms, respectively.

**Figure 10:**
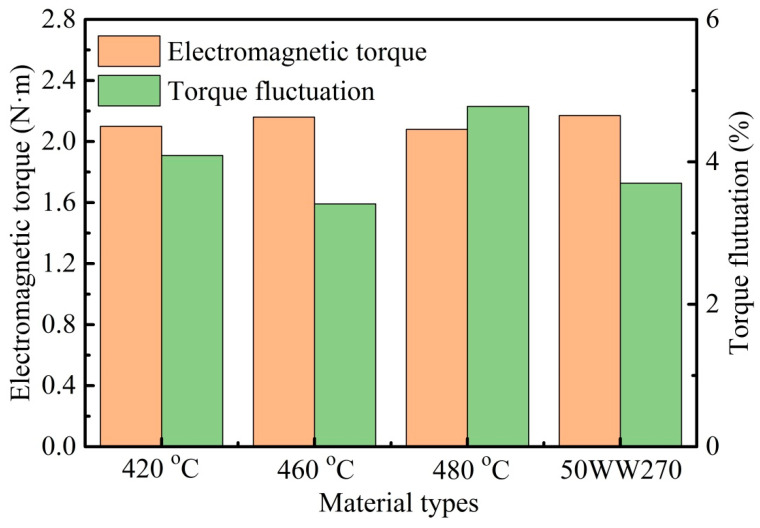
Electromagnetic torque and its fluctuation of motor using different soft-magnetic materials.

To comprehend the magnetic response of the amorphous/nanocrystalline cores required for the stator in a motor, the magnetic flux density cloud of the motor using different soft-magnetic alloys under rated operating conditions is analyzed. Here, the amorphous core annealed at 460 °C and SS are comparatively given in Figure 9, with the motor running times being 5 and 30 ms. As shown in Figure 9a,b, when the motor initially starts and runs for 5 ms, the magnetic flux density is mainly concentrated at the tip of the teeth (red regions), while other areas such as the yoke are less dense, a fact widely accepted in the literature [11,14]. The current of the motor during startup is approximately 4–7 times that of the rated current. The magnetic motive force generated by the stator winding is directly proportional to the current passing through it. Thus, the enhancement of the magnetic motive force leads to a highly saturated state of the amorphous core part of the leakage magnetic circuit. This may be one of the reasons for the high loss of the motor when it first starts. After running for 30 ms, as shown in Figure 9c,d, the magnetic flux density in the vicinity of the tip of the teeth seems to be strengthening, while in the yoke, the phenomenon of supersaturation has not yet been observed, indicating a good operating state. Furthermore, three random locations (m1, m2, and m3) on the yoke of the motor are marked to analyze the magnetic flux density distribution. After running, the locations of m4, m5, and m6 correspond to m1, m2, and m3, respectively, and the corresponding magnetic flux density is listed in Table 2. A slight difference at the marked points is observed. As the motor starts running (5 ms), the magnetic flux density at these points first increases and then decreases, and the maximum values of *B*_s_ are achieved at 460 °C, being the closest to that of the SS motor. This trend is similar to the one observed at 30 ms, indicating that both the amorphous motor at the current temperature and the SS motor exhibit a faster rate of field establishment, which suggests an improved response speed of the motor. To further evaluate the stability of the motor operation, the electromagnetic torque and its fluctuations are studied, as shown in Figure 10. When the motor runs stably, the present amorphous core annealed at 460 °C and the SS exhibit the larger electromagnetic torque in all listed cores, which are 2.16 and 2.17 N·m, respectively, indicating a greater running power and a higher speed of the motor using the present amorphous core and SS. Nevertheless, the lower torque fluctuation of 3.41% for the present amorphous core than that of 3.7% for the SS is achieved. From the stator perspective, smaller fluctuations may reduce the noise and vibration generated during motor operation, thereby improving the operational stability of the motor. All the above experimental and finite-element simulation studies reveal the importance of stator material optimization and provide a theoretical basis for the design and development of amorphous/nanocrystalline motors.

## 4. Conclusions

In this work, the magnetic characteristics of FeSiB amorphous alloys and their influence on motors were systematically investigated via both experiment and finite-element simulation. The main results are summarized as follows.

(1) The FeSiB amorphous core annealed at 460 °C for 10 min exhibits a high magnetic flux density of 1.54 T and low core loss of 0.11 W/kg at 50 Hz and 5.45 W/kg at 1 kHz at 1 T. These values are essential for ensuring high magnetic response and energy efficiency in motors and other electronic devices.
(2) Finite-element simulation analysis reveals that the reduced core loss in the FeSiB motor, compared to the 50WW270 silicon steel motor, is primarily attributable to the stator loss, which in turn is ultimately governed by the microstructure and magnetic properties of the soft-magnetic material.
(3) Both amorphous alloy and silicon steel materials contribute to enhanced motor response speed. The current amorphous motor demonstrates stable electromagnetic torque (2.16 N·m) with reduced torque fluctuation (3.41%), resulting in decreased noise and vibration during motor operation, which improves the motor’s operational stability.

## Figures and Tables

**Table 1:** Main parameters of motor model.

Parameters	Values	Parameters	Values
Rated power/W	750	Pole numbers	10
Rated speed/rpm	3000	Slot numbers	9
Rated voltage/V	110	Rotor inside diameter/mm	10
Length of core/mm	56.7	Rotor outer diameter/mm	39.8
Stator inside diameter/mm	41.1	Connection type	Wye
Stator outer diameter/mm	76	Permanent magnet (PM)	NdFeB

**Table 2:** The magnetic flux density of the different stators after operating for different times.

Operation Time	Position	Types of Stator Materials			
		420 °C (T)	460 °C (T)	480 °C (T)	50WW270 (T)
5 ms	m1	0.79	0.89	0.79	0.96
	m2	0.80	0.82	0.78	0.86
	m3	0.63	0.66	0.60	0.66
30 ms	m4	0.81	0.85	0.81	0.86
	m5	0.73	0.78	0.72	0.79
	m6	0.68	0.69	0.67	0.75

## Data Availability

The original contributions presented in this study are included in the article. Further inquiries can be directed to the corresponding authors.
